# Cardiometabolic risk factors, metabolic syndrome and pre-diabetes in adolescents in the Sierra region of Ecuador

**DOI:** 10.1186/s13098-017-0224-2

**Published:** 2017-04-19

**Authors:** Sharon L. Casapulla, Cheryl A. Howe, Gabriela Rosero Mora, Darlene Berryman, Mario J. Grijalva, Edgar W. Rojas, Masato Nakazawa, Jay H. Shubrook

**Affiliations:** 1 Department of Family Medicine, Ohio University Heritage College of Osteopathic Medicine, 126 Irvine Hall, Athens, OH 45701 USA; 2 Office of Rural and Underserved Programs, Ohio University Heritage College of Osteopathic Medicine, Athens, OH USA; 3 School of Applied Health Sciences and Wellness, College of Health Sciences and Professions, Ohio University, Athens, OH USA; 4 The Diabetes Institute, Ohio University Heritage College of Osteopathic Medicine, Athens, OH USA; 5 Antiviral Research Center, University of California, San Diego, Athens, OH USA; 6 Nutrition Department, College of Nursing, Pontifical Catholic University of Ecuador, Quito, Ecuador; 7 Infectious and Tropical Disease Institute, Department of Biomedical Sciences, Ohio University Heritage College of Osteopathic Medicine, Athens, OH USA; 8 Center for Infectious and Chronic Disease Research, School of Biological Sciences, Pontifical Catholic University of Ecuador, Quito, Ecuador; 9 Department of Primary Care, Touro University College of Osteopathic Medicine, California, Vallejo, CA USA; 10 Department of Surgery, Medical and Social Sciences, University of Alcalá, Madrid, Spain

## Abstract

**Background:**

Excess weight (overweight and obesity) is the major modifiable risk factor for type 2 diabetes mellitus (T2DM) and other non-communicable diseases. However, excess weight may not be as predictive of diabetes risk as once thought. While excess weight and other obesity-related non-communicable diseases are of growing concern in low-middle income countries in Latin America, there is limited research on risk factors associated with T2DM in adolescents. This study investigated prevalence of overweight, obesity, prediabetes, diabetes and metabolic syndrome in adolescents in Ecuador.

**Methods:**

A cross-sectional study was conducted with 433 adolescents from two schools in a small urban center in southern Ecuador and two schools in a large urban center in Quito. Risk factors were measured, including: height, weight, BMI, waist-to-hip ratio, fasting glucose, lipid panel, and HbA1c. Multivariate analysis of variance (MANOVA) was separately applied to risk factors and demographic factors as a set of dependent variables with sex, location and their interaction included as predictors. An independent *t* test was run on the data at 95% confidence intervals for the mean difference. The values for the triglycerides, LDL and VLDL were positively skewed. A Mann–Whitney U test was run on these data.

**Results:**

Using IOTF standards, 9.8% were overweight and 1.9% were obese. Only 1.6% of the sample met the criteria for prediabetes by fasting glucose but 12.4% of the sample met the criteria for prediabetes by HbA1c. None of the participants met criteria for diabetes. There were 2.3% of the participants that met the IDF criteria for metabolic syndrome. Adolescents from the larger urban center had higher rates of prediabetes, higher mean HbA1c, blood pressure, lipid values, and lower HDL levels.

**Conclusions:**

Use of HbA1c identified more adolescents with prediabetes than FBG. The HbA1c measure is an attractive screening tool for prediabetes in developing countries. Although rates of obesity in Ecuadorian adolescents are low there is significant evidence to suggest that prediabetes is permeating the smaller urban centers. Traditional screening tools may underestimate this risk.

## Background

In Latin America (LA), non-communicable diseases (NCDs), including diabetes and cancers, are now among the leading causes of mortality [1]. For example, rates of diabetes in LA are predicted to increase by more than 59%, with 39.9 million predicted to have diabetes by 2030 [2–4]. Excess body weight (overweight and obesity) has been considered the key modifiable risk factor for type 2 diabetes mellitus (T2DM) and represents a major public health concern both in terms of individual quality of life and cost to health care systems in LA [1, 5]. Relatively recent data (2010) suggest that about half of the adults in LA are overweight or obese, up from 33.9% a decade earlier [6]. Adolescents in LA are not immune to this trend. In fact, the World Health Organization (WHO) is calling childhood obesity “one of the most serious public health challenges of the twenty first century [1]”. Countries undergoing rapid demographic and dietary shifts due to an evolving economic climate, such as many countries in Latin America, are among the most vulnerable [1, 6]. A study in adolescent girls in low middle income countries (LMICs) found that LA and the Caribbean had the highest regional prevalence of overweight in both rural and urban areas [7]. Further, it was recently found that the worldwide prevalence of diabetes may be significantly underreported. The International Diabetes Federation previously estimated that 420 million people have diabetes worldwide but it is believed that it is closer to 520 million people [8].

Ecuador is experiencing rapid socioeconomic and demographic transition [9]. Few studies have investigated how these changes have affected the adolescent population in Ecuador in terms of body weight, comorbidities, and cardiometabolic risk factors. Census data indicate that the prevalence of overweight in adolescents (aged 12–19) is 18.8%, obese 7.1%, overweight and obese (combined) is 26% [10] and that prevalence rates of overweight and obesity in adolescents vary by region [10]. The urban Sierra (22.4%), rural Sierra (16.8%) and the rural coastal (15.2%) regions have the highest prevalence of overweight in adolescents. The highest prevalence of adolescent obesity is found in the Galapagos Islands province (13.8%). Rural areas in the costal and Sierra regions had the lowest rates of adolescent obesity (4.2%). Quito, the capital, has prevalence rates of overweight and obese adolescents of 18.8 and 5.5%, respectively [10].

Yepez et al. [11] estimates that the prevalence of overweight and obesity were 13.7 and 7.5%, respectively, in a group of Ecuadorian adolescents. Ochoa-Aviles et al. [12] reports overweight and obesity rates to be 18.0 and 2.1%, respectively. Adolescents from rural areas were 2 to 4-times more likely to present with higher levels of cholesterol, triglycerides, and lower levels of HDL than those from the urban areas. Collectively, these studies demonstrate the need for further investigation into the health of Ecuadorian adolescents [12].

The current study was designed to expand on the previous research to identify and more fully understand the presence of overweight and obesity rates, cardiometabolic risk factors, prevalence of type 2 diabetes, prediabetes and metabolic syndrome in Ecuadorian adolescents. Cariamanga, a small urban center in the southern Loja province, and Pomasqui, a larger urban area within the Quito Metropolitan District are the two areas included in the study. We hypothesized that adolescents from the smaller urban center of Cariamanga would have lower rates of obesity, metabolic syndrome and diabetes, and fewer cardiometabolic risk factors than adolescents living in the larger urban center of Pomasqui.

## Methods

### Study area

A cross-sectional study was conducted from March to July 2015 in two urban centers in the Sierra region of Ecuador. A total of four schools were used in this study, two from larger and two from smaller urban regions. Participants were recruited from two high schools located in Pomasqui, a densely populated urban Parrish belonging to the Quito Metropolitan District in the northern part of the country. Pomasqui (population 28,910) is located along a major highway that connects Quito, the country’s capital (population 1.6 million), with the Coastal region. Agricultural activities are limited due to dry climate. Most of the population works within the Metropolitan district in commerce, manufacturing and service jobs. They are, for the most part, low to low-middle income.

Participants were also recruited from two high schools in the smaller urban center of Cariamanga (population 13,311) located in Calvas County in the southern Sierra Loja Province. Most economic activity among permanent residents involves commerce and service jobs. Because it is the only urban center in the mostly rural Calvas County, part of the population is involved in agricultural production and the schools attract a transient adolescent population from nearby rural villages. The population is largely of low to low-middle income status.

### Sampling

**Fig. 1:**
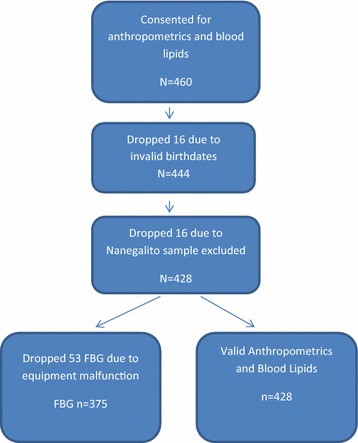
Recruitment of participants, including drop outs at each stage and remaining number of participants

Adolescents ages 13–18 (N = 433) were recruited from two schools in Pomasqui, (n = 219) and two schools in Cariamanga (n = 214). As this was a community- based study and the participants were limited to older adolescents, the participants were not subjected to Tanner staging (See Fig. 1).

### Ethics

The investigative team obtained approval from the local school rectors, the Ministry of Education and the Undersecretary of Education to conduct this study. Adolescents were excluded from the study if they had any physical, physiological, or other health issues (including pregnancy) that would preclude them from participation. A research physician was on site to evaluate any participant if it was unclear whether or not they were appropriate for the study. Parental consent was obtained from parents/guardians, and assent was obtained from all adolescents under the age of 18 years. Informed consent was obtained from all 18-year-old adolescents. All protocols were approved by the Institutional Review Boards of Ohio University and Pontifical Catholic University of Ecuador.

### Anthropometric measurement

Standard procedures were used for assessing anthropometric measures, including light clothing and no shoes. Height was measured to the nearest 0.1 cm using a portable stadiometer (Seca 213 Mobile Stadiometer, Snoqualmie, WA) and weight to the nearest 0.1 kg using a calibrated digital scale (Seca 874 Digital Scale). Height and weight were used to calculate BMI (kg/m^2^). BMI percentile was used to determine weight status according to age and sex-specific growth charts published by the World Health Organization (WHO) [13] and the International Obesity Task Force (IOTF) [14]. Abdominal obesity was assessed using waist-to-hip ratios (WHR = waist/hip). Waist circumference (cm) was measured using a tension-regulated tape measure along a horizontal line at the level of the anterior superior iliac spine. Hip circumference was measured along a horizontal line at maximum posterior extension of the buttocks. All anthropometric measurements were repeated three times by a pair of trained interviewers. Averages of the repeated measurements were used in all calculations and analyses.

### Blood pressure measurements

Systolic blood pressure (SBP) and diastolic blood pressure (DBP) were measured using a manual sphygmomanometer (Welch Allyn DS66, Skaneateles Falls, NY) with an appropriate cuff size. Subjects were seated with their backs and feet supported for at least 5 min before blood pressure was measured. Measurements were repeated two additional times, with a 1–2 min interval between each reading. SBP and DBP measurements were compared to percentile standards for age, sex, and height. Measurements above the 95th percentile were considered hypertensive [15]. Following each BP measurement, resting heart rate (HR) was measured for the last 15 s of 1 min by a trained investigator using the radial pulse. For all measurements, the average of three measurements was used in analyses. Metabolic syndrome was determined using the International Diabetes Federation (IDF) criteria for children and adolescents, grouping those less than age 16 and those age 16 and greater [16].

#### Blood sample analysis

The subjects fasted for at least 8 h prior to participating in the study. Following resting blood pressure measurements, a trained phlebotomist drew intravenous blood samples on site. Blood samples were collected in vacutainer tubes and immediately spun at 3500 rpm for 10 min. Samples were stored in the field at 4 °C until they were transferred to a local lab for analysis within 5 h of collection (Netlab, Quito, Ecuador). Blood samples were analyzed in a laboratory using a method that is NGSP certified and standardized to the DCCT assay for triglyceride (TG) levels, high density cholesterol (HDL), total cholesterol (TC), hemoglobin A1c (HbA1c) and fasting blood glucose (FBG). Very low density lipoprotein (VLDL) and low density lipoprotein (LDL) cholesterol were calculated.

Blood glucose levels were measured with the enzymatic method GOD-PAP [17]. The field research team experienced an equipment error (centrifuge) on one of the testing days in the field. As a result, the FBG readings from 53 participants from the Cariamanga sample were not usable and were excluded from the analyses.

### Data analysis

Multivariate analysis of variance (MANOVA) was separately applied to risk factors (HbA1c, TC, HDL, TG, LDL and VDL) and demographic factors as a set of dependent variables (DVs) with sex, location and their interaction included as predictors. The MANOVA model was followed by a univariate multiple regression model for each DV. If MANOVA F-tests were significant, the univariate models were tested without any adjustment; otherwise, the Holm’s method was applied. Extreme outliers were defined as participants who had standardized residuals greater than 4.5 in any of the variables. All tests were performed 2-tailed at the significance level of 5%. Data were analyzed using IBM SPSS version 22 [18] and the R statistical language version 3.2 [19].

TC, HDL, BMI, FBG and HbA1C were normally distributed for sex groups, and there was homogeneity of variance as assessed by Levene’s Test for Equality of Variances. Therefore, an independent t-test was run on the data as well as 95% confidence intervals (CI) for the mean difference. The values for the TG, LDL and VLDL were positively skewed. Therefore, a Mann–Whitney U test was run on these data.

## Results

A total of 433 adolescents participated in the study (Table 1). The number of adolescents in Cariamanga (49.4%) was similar to the number of adolescents in Pomasqui (50.6%). There were more female (68.1%) than male (31.9%) participants in both locations. Table 2 summarizes descriptive statistics of the overall sample as well as by sex and location. The mean age of all adolescents was 15.1 ± 1.6 years with a mean BMI for age of 20.7 ± 3.4 kg/m^2^, and WHR of 0.83 ± 0.1. Using the IOTF classification system, 9.8% of the total sample was overweight and 1.9% was obese. Table 3 compares results of overweight status and obesity using WHO [13] and IOTF [14] classification systems.

**Table 1:** Frequency distributions by sex and location

	Total		Male		Female	
	*n*	*%*	*n*	*%*	*n*	%
Total sample	433		138	31.9	295	68.1
Location						
Cariamanga	214	49.4	67	31.3	147	68.7
Pomasqui	219	50.6	71	32.4	148	67.6

**Table 2:** Characteristics of sample overall and by location and sex

	Overall			Cariamanga sample									Pomasqui sample								
				All			Female			Male			All			Female				Male	
	n	Mean	SD	n	Mean	SD	n	Mean	SD	n	Mean	SD	n	Mean	SD	n	Mean	SD	n	Mean	SD
Age (years)	433	15.1	1.6	214	14.8	1.5	147	14.8	1.5	67	14.8	1.5	219	15.4	1.6	148	15.4	1.7	71	15.4	1.7
Height (cm)	428	155.7	7.9	214	154.1	7.5	147	152.4	5.3	67	157.8	10.0	214	157.3	7.9	14	154.6	5.7	69	163.2	8.7
Weight (kg)	433	50.4	9.9	214	49.2	9.5	147	49.2	8.5	67	49.1	11.5	219	51.5	10.2	148	50.5	9.4	71	53.5	11.6
Waist (cm)	428	73.8	8.9	214	73.1	8.3	147	74.2	7.5	67	70.7	9.5	214	74.6	9.3	145	74.9	9.5	69	73.9	8.9
Hip (cm)	428	88.8	8.3	214	86.4	7.9	147	87.3	6.8	67	84.2	9.6	214	91.2	7.9	145	92.5	7.5	69	88.6	8.3
WHR	428	0.83	0.05	214	0.85	0.04	147	0.85	0.04	67	0.84	0.05	214	0.82	0.06	145	0.81	0.06	69	0.83	0.05
BMI (kg/m^2^)	428	20.7	3.4	214	20.6	3.2	147	21.2	3.2	67	19.5	3.0	214	20.7	3.6	145	21.1	3.7	69	19.9	3.3
SBP (mmHg)	428	103.3	10.8	214	104.4	10.6	147	103.4	10.4	67	106.6	10.6	214	102.1	10.9	145	100.9	9.7	69	104.7	12.7
DBP (mmHg)	428	69.0	8.5	214	71.0	8.1	147	70.3	7.7	67	72.7	8.8	218	67.2	8.5	147	66.8	8.2	71	68.1	9.2
HR (bpm)	428	76.3	14.4	214	77.8	17.6	147	77.8	17.4	67	77.8	18.1	214	74.8	10.0	145	77.0	9.8	69	70.2	9.0
HbA1c (mg/dL)	427	5.4	0.3	212	5.3	0.3	145	5.3	0.2	67	5.3	0.3	215	5.4	0.3	147	5.4	0.3	68	5.5	0.3
FBG (mg/dL)	375	81.9	9.5	161	79.4	10.4	114	79.2	9.1	67	80.1	13.2	214	83.8	8.4	145	83.0	8.5	69	85.4	7.7
HDL (mg/dL)	428	50.6	14.7	212	46.3	15.7	145	47.9	14.8	67	42.7	16.9	216	54.8	12.3	147	55.8	12.3	69	52.8	12.3
TC (mg/dL)	428	149.6	36.1	212	137.8	39.8	145	145.8	38.1	67	120.4	38.0	216	161.3	27.5	147	165.5	27.8	69	152.2	24.9

	n	Median [interquartile range](range)^a^	n	Median [interquartile range](range)	n	Median [interquartile range](range)	n	Median [interquartile range](range)	n	Median [interquartile range](range)
TG (mg/dL)	428	76 [59–98] (25–367)	145	75 [54–95] (25–367)	67	65 [47–83] (25–202)	147	85 [69–103] (41–285)	69	76 [61–93] (41–302)
LDL (mg/dL)	428	81 [64–98] (17–183)	145	79 [62–99] (24–183)	67	62 [47.5–80] (25–137)	147	89 [76–105.5] (44–161)	69	81 [71–95] (17–142)
VLDL (mg/dL)	428	15 [12–20] (5–73)	145	15 [11–19] (5–7)	67	13 [9.5–17] (5–40)	147	17 [14–21] (8–57)	69	15 [12–19] (8–60)

*WHR* wait-to-hip ratio; *BMI* body mass index; *SBP* systolic blood pressure; *DBP* diastolic blood pressure; *HbA1c* glycated hemoglobin; *TC* total cholesterol; *TG* triglyceride; *HDL* high density lipoprotein; *LDL* low density lipoprotein; *VLDL* very low density lipoprotein; *FBG* fasting blood glucose

^a^Data are represented as mean ± SD, except for TG, LDL, and VLDL who are represented as median [IQR] and (range)

**Table 3:** Percent of sample with each weight class and HbA1c range overall and by location

	Overall		Cariamanga^a^		Pomasqui^a^		Male^b^		Female^a^	
	n	%	n	%	n	%	n	%	n	%
WHO weight classification^c^										
Underweight	31	7.3	14	6.5	17	8.0	17	12.5	14	4.8
Healthy weight	328	76.8	162	75.7	166	77.9	105	77.2	223	76.6
Overweight	41	9.6	22	10.3	19	8.9	9	6.6	32	11.0
Obese	27	6.3	16	7.5	11	5.2	5	3.7	22	7.6
IOTF weight classification										
Thinness grade 3	15	3.5	8	3.7	7	3.3	8	5.9	7	2.4
Thinness grade 2	37	8.7	17	7.9	20	9.4	19	14.0	18	6.2
Thinness grade 1	69	16.2	33	15.4	36	16.9	27	19.9	42	14.4
Healthy weight	256	60.0	125	58.4	131	61.5	73	53.7	183	62.9
Overweight	42	9.8	29	13.6	13	6.1	6	4.4	36	12.4
Obese	8	1.9	2	0.9	6	2.8	3	2.2	5	1.7
HbA1c status										
HbA1c <5.7	374	87.6	200		174		115		259	
HbA1c 5.7–6.4	53	12.4	12		41		20		33	
HbA1c ≥6.5	0	0	0	0	0	0	0	0	0	0

^a^Applying Chi square test, there is no significant difference

^b^Applying Chi square test, there is significant difference P = 0.009 WHO Class and P = 0.002 IOTF Class

^c^Classification using percentiles

Of the 428 samples successfully drawn, a total of 375 adolescents had valid FBG measures. Of these, no adolescents had glucose >126 mg/dL. Six (1.6%) of the adolescents had FBG levels 100–126 mg/dL, indicative of prediabetes. A total of 428 adolescents had a valid HbA1c. None had an HbA1c ≥6.5% (48 mmol/mol), but 53 (12.4%) had an HbA1c 5.7–6.4% (39–46 mmol/mol), indicative of prediabetes.

Ten (2.3%) adolescents (six girls and four boys) over 16 years old met the IDF criteria for metabolic syndrome in children and adolescents, evenly distributed between the two locations. None of the adolescents under 16 met the IDF criteria. In addition to waist circumference, low HDL was the one risk factor common to all ten adolescents who met the IDF for metabolic syndrome. High triglycerides were present in five of the ten adolescents with metabolic syndrome. Elevated glucose was present in only one of the ten. Four (two girls and two boys) of the ten with metabolic syndrome also had prediabetes based on HbA1c.

### Girls versus boys

As expected, there were some significant differences in anthropometric data between boys and girls. Table 3 presents the percentages of overweight and obese by WHO and IOTF classification for both males and females. Girls had higher BMI compared to boys (*p* < 0.001). Boys had higher SBP (*p* = 0.003) and DBP (*p* = 0.05) than girls. Girls had higher TC (*p* < 0.001), LDL (*p* < 0.001), HDL (*p* = 0.01), VLDL (*p* = 0.01) and TG (*p* = 0.009) than boys. There were no significant sex differences in HbA1c (*p* = 0.32). Of the adolescents 16 and older, 33% (39/119) girls had a waist circumference ≥80 cm, and 12% (6/51) boys had a waist circumference ≥90 cm.

### Location differences

Using the IOTF weight classification system, 9.8% of the total sample was overweight, and 1.9% was obese. Total overweight and obesity rates combined were 14.5% in Cariamanga and 8.9% in Pomasqui based on IOTF criteria. Adolescents from Pomasqui had similar rates of obesity (2.8%) to adolescents from Cariamanga (0.9%). While there were no significant differences in rate of obesity by location, adolescents from the smaller urban center of Cariamanga did have higher rates of overweight (13.6%) than adolescents from the larger urban center of Pomasqui (6.1%), using the IOTF criteria. Adolescents from Cariamanga had higher WHRs (0.85 ± 0.04) than (0.82 ± 0.06) adolescents from Pomasqui (*p* < 0.001). There was a higher proportion of overweight adolescents in Cariamanga than in Pomasqui (*p* = 0.018) but no significant difference in obese adolescents (*p* = 0.30).

Adolescents from Pomasqui had higher prevalence of prediabetes (HbA1c 5.7–6.5%; 39–46 mmol/mol) (16.8%) than adolescents from Cariamanga (1.9%) (*p* < 0.001) and higher FBG (*p* < 0.001) than adolescents Cariamanga. Adolescents from Pomasqui also had higher TC (*p* < 0.001), LDL (*p* < 0.001), VLDL (*p* = 0.02), and TG (*p* = 0.03). In contrast, HDL was lower in adolescents from Cariamanga (*p* < 0.001). Adolescents from Pomasqui had higher SBP (*p* = 0.03) and DBP (*p* < 0.001).

### Sex by location interaction

Both sex and location were significant predictors of the six cardiometabolic risk factors (HbA1c, TC, HDL, TG, LDL and VLDL) as a set [sex: Wilk’s λ = 0.93, F(6, 418) = 5.2, *p* < 0.001; location: Wilk’s λ = 0.87, F(6, 418) = 10.1, *p* < 0.001], while their interaction was not (Wilk’s λ = 0.99, F(6, 418) = 0.8, *p* > 0.05). Specifically, sex was associated with all risk factors (*ps* < 0.05) except for HbA1c (*p* > 0.05) after controlling for location. Location was significantly associated with all risk factors (*ps* < 0.05) after controlling for sex. The location by sex interaction was not significant for any of the risk factors (*ps* > 0.05).

Table 4 summarizes the results of the regression models, showing how sex and location influenced the prevalence of the cardiometabolic risk factor. All risk factors, except for HbA1c, were significantly associated with both sex and location. Specifically, boys had lower levels of TC, HDL, LDL, TG and VLDL than girls regardless of location (*ps* < 0.05). Adolescents from Pomasqui had higher values of all risk factors, regardless of sex (*ps* < 0.05). Sex and location did not interact to influence any of the cardiometabolic risk factors (*ps* > 0.5).

**Table 4:** Regression analyses of the cardiometabolic risk factors, anthropometrics and hemodynamics

	(Intercept)	Sex (male)	Location (urban)	Sex x location
HbA1c (units)	5.28 ± 0.02***	0.04 ± 0.04	0.15 ± 0.03***	−0.01 ± 0.05
FBG (mg/dL)	79.17 ± 0.87***	0.90 ± 1.61	3.85 ± 1.16**	1.49 ± 2.10
TG (mg/dL)	82.17 ± 3.23***	−13.11 ± 5.74*	9.5 ± 4.55*	5.66 ± 8.07
TC (mg/dL)	145.8 ± 2.73***	−25.4 ± 4.86***	19.72 ± 3.85***	12.04 ± 6.84
HDL (mg/dL)	47.94 ± 1.16***	−5.27 ± 2.06*	7.82 ± 1.63***	2.26 ± 2.9
VLDL (mg/dL)	16.46 ± 0.65***	−2.61 ± 1.15*	1.85 ± 0.91*	1.11 ± 1.62
LDL (mg/dL)	81.58 ± 2.09***	−16.52 ± 3.72***	9.86 ± 2.95***	7.64 ± 5.23
Waist (cm)	74.22 ± 0.72***	−3.49 ± 1.29**	0.68 ± 1.03	2.47 ± 1.82
Hip (cm)	0.85 ± 0.0***	−0.01 ± 0.01	−0.04 ± 0.01***	0.03 ± 0.01***
BMI (kg/m^2^)	21.16 ± 0.28***	−1.68 ± 0.49***	−0.1 ± 0.39	0.51 ± 0.69
SBP (mmHg)	103.44 ± 0.88***	3.11 ± 1.57*	−2.59 ± 1.24*	0.76 ± 2.2
DBP (mmHg)	70.26 ± 0.68***	2.42 ± 1.22*	−3.46 ± 0.97***	−1.15 ± 1.71

Data are expressed as β ± standard error

*WHR* wait-to-hip ratio; *BMI* body mass index; *SBP* systolic blood pressure; *DBP* diastolic blood pressure; *HbA1c* glycated hemoglobin; *TC* total cholesterol; *TG* triglyceride; *HDL* high density lipoprotein; *LDL* low density lipoprotein; *VLDL* very low density lipoprotein; *FBG* fasting blood glucose

* *p* < 0.05, ** *p* < 0.01, *** *p* < 0.001

The multivariate F-test revealed that waist circumference, WHR, BMI, as well as SBP and DBP were significantly affected by both sex and location as well as their interaction [sex: Wilk’s λ = 0.95, F(5, 420) = 4.8, *p* < 0.001; location: Wilk’s λ = 0.72, F(5, 420) = 32.6, *p* < 0.001; interaction: Wilk’s λ = 0.97, F(5, 420) = 2.9, *p* = 0.01). Specifically, sex was significantly associated with waist, BMI, SBP, and DBP (*ps* < 0.05) after controlling for location, while location alone was associated with WHR, SBP, and DBP (*ps* < 0.05). Sex and location interacted to affect WHR only (*p* < 0.001). In Cariamanga, boys and girls did not differ in WHR (*p* > 0.05), while in the Pomasqui, boys had higher WHR (*p* < 0.001). Conversely, girls from Cariamanga had higher WHR than girls from Pomasqui (*p* < 0.001) while boys did not differ between locations (*p* > 0.05). Regarding the other variables, waist, BMI, SBP, and DBP were all affected by sex (*p* < 0.05). Specifically, boys had lower waist and BMI, but higher SBP and DBP measures than girls, regardless of location. Adolescents from Pomasqui had lower SBP and DBP than their counterparts from Cariamanga (*ps* < 0.05) regardless of sex.

## Discussion

There were several notable findings from this study. First, the prevalence of overweight and obesity in the overall sample were lower than anticipated based on previous research in this region [11, 12]. Second, there were relatively low rates of biochemical metabolic abnormalities (dyslipidemia and hyperglycemia) and only 2.3% met the IDF criteria for metabolic syndrome. Third, despite these factors, almost 10% of the adolescents (n = 43) in the sample met the diagnostic criteria for prediabetes. Finally, the rate of prediabetes was eight times higher in adolescents from the large urban center of Pomasqui as compared to the smaller urban center of Cariamanga.

Based on census data and recent studies mentioned previously, higher rates of metabolic abnormalities (dyslipidemia and hyperglycemia) were expected in the overall sample. Only 2.3% of adolescents (n = 10) met the IDF criteria for metabolic syndrome. The higher rates of cardiometabolic risk factors in the larger urban center of Pomasqui may reflect the availability of a more westernized diet, as has been suggested in previous research in LMICs [5]. However, it was surprising that the rates of overweight were higher in the smaller urban areas of Cariamanga (13.6%) compared to the larger urban center of Pomasqui (6.1%). The authors believe that the findings demonstrate that the problem of excess weight is spreading to smaller communities in Ecuador and that these more rural regions, farther from larger urban centers, are also experiencing the early stages of nutrition transition.

Nearly 10% of adolescents in the sample had prediabetes based on an elevated HbA1c and rates were 8× higher in the larger urban center of Pomasqui (16.8 vs 1.8%). Both the differing rates of prediabetes and the higher rates of overweight may indeed confirm that Ecuadorian adolescents are in the midst of a nutrition transition [12]. These results may indicate that the effects of a western lifestyle and diet are permeating more of the country, even into the smaller and more remote urban areas in southern Ecuador [12]. Of note, the American Diabetes Association recommends screening any person above the age of 10 who is obese and has at least 2 additional risk factors for T2DM. This screening can be completed using an A1c [20]. If screenings in this study were limited to this standard, a much smaller number of adolescents would have been screened and many cases of prediabetes would have been missed.

Previous research in the United States showed that the HBA1c may be less sensitive than an oral glucose tolerance test (OGTT) to diagnose prediabetes and diabetes [21]. However, OGTT are often not practical in rural and underserved areas. The use of an OGTT has almost been completely replaced by the A1c in adults. Because of these challenges some authors have suggested that the A1c is a necessary tool for the screening of dysglycemia in adolescents [22]. A recent study actually showed that current A1c cutoffs may underestimate diabetes rates in adolescents [23].

The results of this study have important clinical implications for screening adolescents. Use of HbA1c identified more adolescents with prediabetes than FBG and other metabolic markers. This has also been reported in adults [24]. The HbA1c measure is an attractive screening tool for prediabetes in developing countries for several reasons: (1) it does not require the person to fast, (2) it is available as a venous or capillary sample, and (3) it is enzymatically easier to process than glucose [25]. Clearly, if a more traditional biochemical risk factors were used in this present study, fewer adolescents would have been identified as at risk. Use of HbA1c could be used as a screening tool to provide identification earlier on the insulin resistance spectrum. The importance of early identification in adolescence cannot be understated, as it is now known that type 2 diabetes in childhood and adolescence is less responsive to treatment and that adolescents experience beta cell failure faster than others with type 2 diabetes [26].

This study has a number of limitations. While this is a representative sample of adolescents in Quito and the southern Sierra region of Ecuador, it does not represent the diversity of adolescents in the country. Geographic differences such as elevation could be confounding variables affecting some of our results. The study team did not do Tanner staging on the participants which limits the confidence that we only included mature adolescents. Further, we did not assess participants for anemia and cannot rule out that some of the participants had a hemoglobinopathy. However, this would have resulted in an under-estimation of diabetes rates. In this study socioeconomic data were not collected, and as suggested by previous research [12, 27, 28], socioeconomic factors could also be influencing our results. Finally, this study measures a single point in time, and cannot predict the trajectory of these adolescents based on this measurement alone. In light of the higher rates of overweight in the adolescents from the smaller urban center, longitudinal follow up would be illustrative. The use of HbA1c is also somewhat controversial. The OGTT is the gold standard but in the U.S it has almost entirely been replaced by the HbA1c due to the ease of testing.

Difficulties experienced in this study include not having longitudinal access to the study subjects and variability in the local resources. Due to inconsistent electricity, we lost a substantial number of glucose readings because centrifugation of the sample was inadequate and glycolysis occurred in the sample vials. Despite the limitations, this study exposes some important differences that need further investigation in order to identify those adolescents at greatest risk of chronic diseases. Additional studies to determine specific lifestyle factors that influence these risk factors are currently being conducted with adolescents in Ecuador. Future studies should continue this work to determine what physiologic or environmental features best predict progression to or prevention of diabetes. In the meantime strong consideration should be placed on using the HbA1c for screening for diabetes and prediabetes in LA adolescents.

## Conclusions

In conclusion, this study found lower than anticipated rates of overweight and obesity as well as relatively low rates of biochemical metabolic abnormalities and metabolic syndrome. Notably, 10% of the adolescents in the sample met the diagnostic criteria for prediabetes, despite low levels of dyslipidemia and hyperglycemia. Although rates of obesity in Ecuadorian adolescents are low there is significant evidence to suggest that prediabetes is permeating the smaller urban centers. Perhaps of most concern, and worthy of future study, is the clear and substantive disparity in rates of prediabetes in adolescents from the large urban center as compared to the smaller urban center. Use of HbA1c identified more adolescents with prediabetes than FBG. The HbA1c measure is an attractive screening tool for prediabetes in developing countries. Traditional screening tools may underestimate this risk.

This study builds upon important research describing the epidemiology of children and adolescents in developing countries in LA. Developing countries with public health systems that can recognize and respond to these health challenges in the adolescent population may be more able to slow the rise of many preventable chronic diseases such as diabetes. The non-communicable chronic diseases such as diabetes, hypertension, and non-alcoholic fatty liver disease will become an increasing burden for countries in transition if no action is taken to reduce the precursors of these diseases in children and adolescents.
